# Non-random genomic integration - an intrinsic property of retrogenes in Drosophila?

**DOI:** 10.1186/1471-2148-10-114

**Published:** 2010-04-28

**Authors:** Muralidhar Metta, Christian Schlötterer

**Affiliations:** 1 Institut für Populationsgenetik, Vetmeduni vienna, Veterinärplatz 1, 1210 Wien, Austria; 2 Unit of Animal Genomics, GIGA-R, B34 +1, University of Liège, 4000 Liège, Belgium

## Abstract

**Background:**

The Drosophila X-chromosome shows a significant underrepresentation of genes with male-biased gene expression (demasculinization). This trend is matched by retrogenes, which typically have a male biased gene expression pattern and show a significant movement bias from X-chromosomes to autosomes. It is currently assumed that these patterns are best explained by selection, either mediated by male meiotic sex chromosome inactivation (MSCI) or sexually antagonistic forces. We scrutinized the evolutionary dynamics of retroposition by focusing on retrogenes for which the parental copy has degenerated.

**Results:**

Consistent with a functional substitution of the degenerated gene by the retrogene, patterns of sequence evolution and gene expression were similar between retroposed and parental genes. Like previous studies, our set of retrogenes showed a significant movement off the X-chromosome. In contrast to data sets where retroposition caused gene duplication, the genes in our study showed primarily female-biased or unbiased gene expression.

**Conclusions:**

Based on our results, the biased transposition pattern cannot be explained by MSCI and probably not by sexual antagonism. Rather, we propose that the movement away from the X-chromosome represents a general property of retroposition in Drosophila.

## Background

The integration of reverse transcribed messenger RNA into the genome is called retroposition. This process has important evolutionary consequences as it typically results in an additional gene copy, lacking introns and regulatory sequences. In Drosophila retrogenes show interesting dynamics. Rather than moving randomly in the genome, retroposed gene copies preferentially originate from X-linked genes and move to autosomes [1]. In combination with the preferential male biased gene expression of retroposed genes, their non-random integration pattern has been used to explain the "demasculinization of the X-chromosomes" [2,3]. In comparison to autosomes, X-chromosomes contain fewer genes that are more strongly expressed in males than in females [4]. This underrepresentation of male biased genes on the X-chromosome is not yet fully understood. It has been suggested that male meiotic sex chromosome inactivation (MSCI) or sexual antagonism may be the evolutionary force driving the movement of retroposed genes, which ultimately leads to contrasting patterns in gene expression between X-chromosome and autosomes [5-7].

Central to this interpretation is the assumption that the parental gene had a functionally important gene expression during male meiosis (i.e.: either a male biased gene or housekeeping gene) [8]. A retroposed copy could substitute the function during male meiosis and thus avoid the deleterious effect of MSCI in testis [9-11]. Given the experimental challenge to disentangle the division of function among the retroposed and parental copy as well as distinguishing between subfunctionalization and neofunctionalization, we used an alternative approach to test the contribution of retroposition to the demasculinization of the Drosophila X-chromosome.

## Results

Aiming for functional similarity of the retrogene and its parental copy, we analyzed only those cases where the parental copy, giving rise to the retroposed gene, had been lost. We used the well-curated data set of positionally relocated genes for 12 Drosophila species [12] to identify bona fide retroposed genes. In case of retrotransposition, the copy without introns is the retroposed copy while the copy with introns is the ancestral copy. Hence we used the following criteria: 1) absence of introns in at least one species, and 2) presence of at least one intron in at least one species. This resulted in 46 genes that were relocated by retroposition and for which the parental copy was degenerated/lost. We further scrutinized this data set by keeping only those genes for which at least one flanking gene confirmed the chromosomal relocation. We also eliminated genes from genomes with low sequence coverage that exhibited very poor sequence quality. After these quality control steps, we obtained 20 genes that unambiguously relocated by retroposition and for which the parental copy was degraded. This data set was expanded to include the gene *RplP2 *(CG4918) for which we had independent evidence from SAGE data [13,14] (see additional file 1). Hence, in total our data set consisted of 21 genes with an unambiguous relocation of the retroposed copy while the parental copy was degraded.

All 21 genes were preserved over 40 million years (i.e., present in *D. melanogaster *and *D. grimshawi*), suggesting the functional importance of these genes. Table 1 show that not all genes are annotated in 12 Drosophila species. Nevertheless, for all genes except CG16771 and CG14286, the gene could be identified at least in a close relative, suggesting that the missing annotations in FlyBase reflect imperfections of the available genomic sequences rather than a loss of genes. Moreover, BLAST search of *D. melanogaster *protein against other sequenced Dipteran insect genomes *Culex pipens, Aedes aegypti *and *Anopheles gambiae*, covering approximately about 250 million years of divergence, suggests their conservation in other Dipteran species (see table in additional file 2).

**Table 1:** List of genes that lost their parental copy and the location on Muller's element in different species

	Dmel	Dsec	Dsim	Dyak	Dere	Dana	Dpse	Dper	Dwil	Dmoj	Dvir	Dgri
CG11164	A	A^†^	A	A	A	A	A	A	A	***C***	***C***	***C***
CG11790	E	E	E	E	E	E	E^†^	E	E	***B***	***B***	***B***
CG12375	***B***	***B***	***B***	***B***	***B***	***B***	A	A	A	A	A	A
CG1354	A	A	A	A	A	***D***	A	A	A	A	A	A
CG14286	***E***	***E***	***E***	***E***	***E***	-	A^†^	A	A	A	A	A
CG14618	A	A	A	A	A	A	A	A	A	***C***	***C***	***C***
CG14779	A	A	A	A	A	***E***	A	A	A	A	A	A
CG1639	A	A	A	A	A	A	***E***	***E***	A	A (C)^‡^	A (C)^‡^	A (C)^‡^
CG16771	***B***	***B***	***B***	***C/B***	***C/B***	***B***	-	-	A	A	A	A
CG2059	A	A	A^†^	A	A	***B***	A	A	A	A	A	A
CG2227	***A***	***A*^†^**	***A***	***A***	***A***	***A***	***A***	***A***	***A***	E	E	E
CG32441	D	D	D	D	D	D	D	D	D	***E***	***E***	***E***
CG33250	A	A	A	A	A	***D***	X^†^	X^†^	A	A	X^†^	A
CG4918	***E***	***E***	***E***	***E***	***E***	A	A	A	A	A	A	A
CG5029	B	B	B^†^	B	B	B	B^†^	B	B	***A***	***A***	***A***
CG6284	***E***	***E***	***E***	***E***	***E***	***E***	B	B^†^	B	B	B	B
CG8239	A	A	A	A	A	A	A	A	A	***C***	***C***	***C***
CG8939	A	-	A	A	A	***D***	X	X	A	A	A	A
CG9126	A	A	A	A	A	***D***	X	X	A	A	A	A
CG9172	***A***	***A***	***A***	***A***	***A***	***A***	***A***	***A***	***A***	C	C	C
CG9742	A	A	A	A	A	A	***E***	***E***	A	A	A	A

Dmel:*D. melanogaster*; Dsec:*D. sechellia*; Dsim:*D. simulans*; Dyak: *D. yakuba*; Dere: *D. erecta*; Dana: *D. ananassae*; Dpse: *D. pseudoobscura*; Dper: *D. persimilis*; Dwil: *D. willistoni*; Dmoj: *D. mojavensis*; Dvir: *D. virilis*; Dgri: *D. grimshawi*; Bold and italicized font indicates retroposed copy; 'X' mark indicates that the chromosomal location is not assigned due to lack of flanking genes on the scaffold on which the gene is located; Muller's element A corresponds to the X-chromosome in all species. In *D. pseudoobscura*, *D. persimilis*, *D. willistoni*, Muller's element D represents XR chromosome. All other elements represent autosomes; '-' indicates that there is no hit for the gene in the corresponding species; ^†^The gene is not fully predicted in the respective species due to bad quality of the sequence; ^‡^The gene CG1639 in these species possess two copies; Parental copy on the Muller's element A and retro copy on the Muller's element C. For the same gene, degenerated parental copies exist for species *D. pseudoobscura *and *D. persimilis *on Muller's element A.

Retroposition has frequently been shown to result in an accelerated rate of evolution, probably reflecting the acquisition of novel functional properties [15]. We tested for evidence of accelerated evolution after the retroposition event using the branch model of PAML [16]. Out of 20 genes tested, we found four genes with a *p*-value smaller than 0.05 (CG2059, CG2227, CG12375, CG14286), but after Bonferroni correction for multiple testing none of them remained significant (see table in additional file 3). Hence, we did not find evidence for a functional shift of the retrogenes as evidenced by accelerated sequence evolution after the transposition event.

As a final test for functional equivalence of retroposed and parental copies we compared sex-biased gene expression (Table 2). About 40% of the genes (CG12375, CG1354, CG14779, CG16771, CG2059, CG33250, CGCG4918, CG8239, CG9126) showed the same sex-bias among all species, irrespective of whether a retroposed gene or the parental gene was present in the corresponding species. Hence, all these genes had an expression pattern that was similar between retroposed and parental gene copies. Several genes showed, however, a striking heterogeneity in gene expression among species. Interestingly, this heterogeneity in gene expression could be detected among species carrying the retroposed copy as well as among species carrying the parental copy. Hence, the sex-biased gene expression pattern of these genes is highly unstable, but no apparent effect of the retroposition could be noted. Taken together, the results of gene conservation, analysis of molecular evolution and gene expression suggest that transposition did not result in a functional alteration. Retroposed and parental copies are therefore probably functionally equivalent.

**Table 2:** Sex-biased gene expression of the genes based on microarray analysis in different Drosophila species [2]

	Dsim	Dyak	Dana	Dpse	Dvir	Dmoj
CG11164	-0.396*	-0.214	-0.366	-0.369	**-0.431**	**-0.449**
CG11790	0.652	-0.198	-0.139	-1.116*	**-0.209**	-0.822*
CG12375	**0.062**	**-0.065**	**-0.248**	-0.486	-0.181	0.077
CG1354	-0.471*	-0.597*	-0.358*	-0.629*	-0.988*	-0.636*
CG14286	**0.378**	**-0.505**	-	0.076	-0.457	-0.961*
CG14618	-0.184	-0.112	-0.064	0.000	**-0.149**	-0.431*
CG14779	-0.036	-0.176	**0.116**	-0.244	-0.151	-0.915
CG1639	-0.052	-0.323	0.019	**-0.402**	0.358*	-0.177
CG16771	**0.414**	**0.163**	**-0.043**	-	-0.082	0.037
CG2059	0.251	0.130	**0.039**	0.181	0.154	0.137
CG2227	-0.151*	**0.128**	**-0.130**	**-0.085**	-0.212	-
CG32441	0.713	0.432	0.002	-0.368*	0.587*	**-0.082**
CG33250	-0.068	-0.321	**-0.071**	-0.286	-	-
CG4918	-0.807*	-0.772*	-1.911*	-2.491*	-1.284*	-
CG5029	0.937*	0.349*	-0.550*	-0.361*	**0.140**	0.615*
CG6284	**-0.216**	**-0.223**	**0.040**	-	-0.214	-0.622*
CG8239	-	-0.298	-	-	**0.044**	**-0.323**
CG8939	-0.512*	-0.449*	**-0.398**	-1.489*	-0.401	-0.271
CG9126	-	-0.003	**-0.017**	-	-	-
CG9172	**0.112**	0.331*	-0.842*	-0.610*	-0.164	0.245
CG9742	-0.577	-0.727*	-0.356	-0.618*	-0.258	-0.915*

Dsim: *D. simulans*; Dyak: *D. yakuba*; Dana:*D. ananassae*; Dpse: *D. pseudoobscura*; Dvir: *D. virilis*; Dmoj: *D. mojavensis*; The expression values are the log_2 _ratios of male vs. female intensities. Negative values indicate that the expression is biased towards females while positive values indicate opposite. * indicates a significant sex bias in gene expression. Retroposed copies are indicated by a bold font.

**Figure 1:**
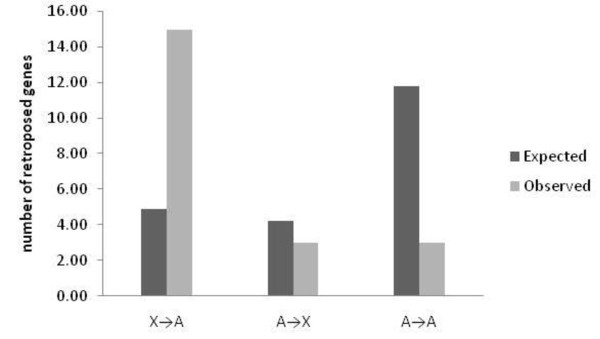
**The pattern of retroposition between sex chromosomes and autosomes among the Drosophila species**.

Out of 21 functionally equivalent retroposition events, 15 genes moved from the X-chromosome to an autosome (Figure 1). In contrast, only three moved from an autosome to the X chromosome and three moved among autosomes. This is a highly significant excess of retroposition events off the X-chromosome (χ^2 ^= 27.884; df = 2; *p *= 8.8 × 10^-7^). This pattern of retroposition has been previously described and explained by selection (mediated either by MSCI or sexual antagonism) favouring an autosomal location of genes with male-biased gene expression [1]. Interestingly, not a single gene in our data set has male biased gene expression in all or the majority of the species analyzed. Apart from CG5029, for which 50% of the species showed male-biased gene expression, significant male-biased gene expression was observed for only three individual gene/species combinations (Table 2). This contrasts with 29 species/gene combinations showing significant female-biased gene expression.

**Figure 2:**
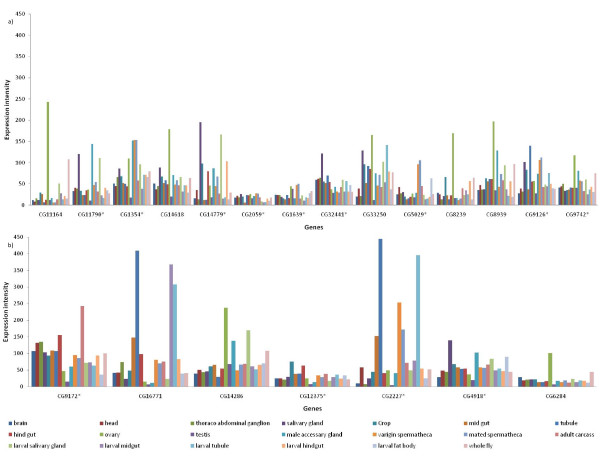
**Gene expression in different tissues at larval and adult stages in D. melanogaster for the genes**. a) with the parental copy b) with the retrocopy [36]. The gene expression intensity for the genes with * mark are reduced by 10-fold for the ease of representation.

Since male biased expression is only a rough measure of testis expression, we further looked at the retroposed copies that are located on the autosomes in *D. melanogaster *for their testis specificity in expression. According to the criteria of [17], only one out of 5 genes was expressed in testis, and even this one had a lower testis expression than in ovaries or most of the other tissues (Figure 2b, additional file 4). Hence, the preferential movement out of the X-chromosome in our data set cannot be explained by selection operating on genes with male-biased or testis expression.

## Discussion

Retroposition creates one additional gene copy, which lacks introns and most of the regulatory sequences. In most cases, the retroposed copy either acquires a novel function (neofunctionalization) [18] or some of the functions of the parental gene are divided among the retrogene and the parental copy (subfunctionalization) [19]. Here we have studied a special case, in which the retrogene has functionally replaced the parental copy that was either degenerated or lost. If the parental gene copy has a very simple expression pattern, it is conceivable that a freshly retroposed gene could by chance capture regulatory sequences that mimic the parental expression pattern. We found considerable gene expression differences among tissues for those genes of our data set, which carry introns in *D. melanogaster *(Figure 2a, additional file 4). Hence, it appears unlikely that a freshly retroposed gene could already fully substitute the parental copy, as it is assumed that retrogenes start with a simple expression pattern and acquire more complex regulation later [20]. Indeed, we found that those genes for which only the retrogene is present in *D. melanogaster *also showed heterogeneity in gene expression among tissues (Figure 2b, additional file 4). Thus, some explanation is required to justify how retrogenes could be maintained given that Drosophila has a pronounced mutation bias towards deletions [21], which is expected to degenerate the retrogene. It is conceivable that the retrogene first acquires a function, either by subfunctionalization [22] or neofunctionalization [23]. If a subsequent mutation in the parental copy causes a slightly impaired function, it is possible that selection could favour the spread of regulatory mutations that restore gradually the function of the parental copy. Once the retrogene is able to substitute the vital functions of the parental copy, the original copy could accumulate further mutations and will be eventually lost. Some support for this model could be gleaned from the genes CG9742 and CG1639, for which we still detected traces of the parental gene in *D. pseudoobscura *and *D. persimilis*, but which had already accumulated several stop codons. Another interesting case is the gene CG1639. In *D. pseudoobscura *and *D. persimilis*, the retrogene has replaced the parental copy. In *D. virilis*, *D. mojavensis *and *D. grimshawi*, we detected a second origin of a retroposed copy, but in this case the parental copy is still preserved. Based on the divergence time of these species, the retrocopy originated about 30-40 million years ago. In addition, it has been shown that in Drosophila *e(y)2*, a retrocopy of *e(y)2b *gene functionally displaced the parental copy during evolution [24]. Similarly, TAF1, a human ubiquitously expressed general transcription factor was shown to be functionally replaced by its retroposed homologue TAF1L [25]. It appears that it is not uncommon that the retrogene and parental gene can have similar function.

The preferential male-biased gene expression of retrogenes in combination with the under-representation of male-biased genes on the X-chromosome has been widely viewed as strong support for selection driving the pronounced movement bias of retrogenes from the X-chromosome to the autosomes. Further insights into the evolutionary fate of male-biased genes could be obtained from the analysis of the Muller's element D, which is ancestrally autosomal, but has been translocated and forms part of a neo-X chromosome in *D. pseudoobscura*. If selection is a major evolutionary force for the movement of retrogenes, one may expect an increased rate of retroposition events off the X-chromosome. Indeed, Sturgill et al. [2] described eight male biased genes that moved from the X to the autosomes. Nevertheless, a re-analysis of the chromosomal movements did not provide support for retroposition driving this relocation. Rather, we found unambiguous support for a genomic translocation for six genes. Hence, despite a presumably strong selective force driving male biased genes off the X-chromosome, we found no evidence that retroposition mediates this effect.

Two recent studies found a general trend for the movement of genes from the X-chromosome to the autosomes and the relocated genes showed an expression in testis [26,27]. While this observation is consistent with X-linked genes escaping inactivation during spermatogenesis, there are some open questions. First, as both copies of the gene continue to be present it remains speculative to what extent the duplicate gene has acquired a function during spermatogenesis from the paternal copy. Second, also genes moving among autosomes show male biased gene expression [27]. Third, one study relied entirely on data from *D. melanogaster *to identify expression in testis [26]. Given the high turnover of sex-biased gene expression it is not clear how robust this approach is [28]. Fourth, it is not yet understood how strong the selection against male biased/testis expressed genes on the X chromosome is. While there is little doubt about the existence of the X chromosome inactivation during spermatogenesis, it is also clear that many genes with an expression in testis persist on the X-chromosome.

Our analysis of retrogenes lacking a functional copy relies on a conceptually different approach to understand the movement of genes away from the X-chromosome. These genes showed the same insertion bias as other retrogenes, but in contrast to previously analyzed retro genes [1] no male-biased gene expression was found. This observation provides no support for a simple selection scenario favouring an autosomal location of male-biased genes. Rather it suggests a neutral mutation bias that prefers the origin of retrogenes on the X-chromosome and their insertion into autosomes. We note, however, that in mammals there is strong evidence that the out of the X-movement is not the product of a simple transposition bias, as pseudogenes do not show this effect [29]. Hence, it may well be that the evolutionary forces responsible for the out of the X-movement differ between Drosophila and mammals. One obvious difference between Drosophila and mammals that could be involved in this difference is the dosage compensation mechanism.

## Conclusions

Consistent with previous results, our data suggests an excess of retrotransposition events out of X-chromosome in Drosophila, but do not show a male-biased or testis specific expression. These results indicate that the biased transposition pattern cannot be due to MSCI or sexual antagonism, rather the pattern is a general property of retrotransposition in Drosophila.

## Methods

### Identification of retrogenes with degenerated parental copies

Drosophila positionally relocated genes [12] were screened for genes that are putatively relocated due to retroposition. 46 genes were identified which are likely to be retrogenes that lost the parental copy in some species. These genes were reannotated using Genescan [30] and GeneWise [31] programs to confirm the gene models in all 11 newly sequenced *Drosophila *species [32]. For identifying the chromosomal location, we used information from the flanking genes, as sequences from all the species were not assigned to particular chromosomes. Using the assemblies of 11 Drosophila species http://flybase.org, we checked for the orthologs of the neighbouring genes in *D. melanogaster *on a scaffold in which the gene is located and used this information for assigning the Muller's element location of the gene in a species. Genes that lack support from flanking genes about their chromosomal assignment and genes that were misclassified due to sequencing artefacts were removed from further analysis (Additional file 5). Out of 46 genes, 26 were ambiguous and were excluded from the analysis. The remaining genes are retrogenes that lack a parental copy in some species (Table 1). For some genes the parental copy in *D. melanogaster *has more than one isoform. We used all isoforms to predict the gene models and all of them show the loss of introns confirming retrotransposition event. To this data we added the gene *RplP2 *which contained introns in the UTR regions (Additional file 1).

### Cross species conservation

To identify the evolutionary conservation of these genes across different Dipteran species that diverged about 250 million years ago, we performed BLASTP search against *Culex pipens*, *Aedes aegypti *and *Anopheles gambiae *genomic sequences.

### Identification of selection

Multiple sequence alignments were obtained using 'Dialign' program [33] and inspected manually for alignment artefacts. A phylogenetic tree for each gene was constructed using the Tree Puzzle program [34]. The lineage separating species with parental and retrogene is considered to be a foreground branch. If the retrocopy evolves at a different rate than the parental copy, this lineage should evolve at an accelerated rate. Branch models as implemented in the PAML program were used for identification of an accelerated rate of evolution. The null hypothesis in the branch model is that all the branches have a single ω (d_N_/d_S_) ratio and was tested against the alternative two-ratio model that allows a different ω ratio for the foreground branch [35]. A significant likelihood ratio test indicates that the foreground branch is evolving at an accelerated rate of evolution.

### Gene expression analysis

The expression in different tissues of larval and adult stages in *D. melanogaster *was obtained from the FlyAtlas database [36]. The sex-biased expression in *D. simulans*, *D. yakuba*, *D. ananassae*, *D. pseudoobscura*, *D. virilis *and *D. mojavensis *was obtained from [28]. A significant sex-bias in gene expression was taken from [28], which was determined based on a Mann-Whitney U test corrected for false discovery rate.

### Expected number of retroposition events between chromosomes

The expected number of retroposition events was obtained using the expectation formula developed by Betran et al [1] in *D. melanogaster*, which accounts for the number of genes per chromosome, size of the chromosome and dosage compensation. A chi-square test was performed to detect heterogeneity among the retroposition events between chromosomes.

## Authors' contributions

MM participated in the design of the study, carried out the experiments, analyzed the data and drafted the preliminary manuscript. CS conceived of the study, and participated in its design and coordination and drafted the manuscript. All authors read and approved the final manuscript.

## Supplementary Material

Additional file 1 CG4918 is a retroposed gene in Drosophila. Click here for file

Additional file 2 **Conservation of the candidate genes in other Dipteran species spanning approximately 250 million years of divergence**. BLAST score and E-values of the genes in other Dipteran species based in default parameters in FlyBase. Click here for file

Additional file 3 Likelihood ratio tests for branch models as implemented in PAML to test the evidence for an accelerated rate of evolution after retrotransposition (foreground lineage). Click here for file

Additional file 4 Gene expression values based on FlyAtlas database [36] in different tissues at larval and adult stages in *D. melanogaster* for the genes with the parental copy and with the retrocopy. Click here for file

Additional file 5 **Screening for putative candidate genes from Bhutkar et al., data**[12]. Click here for file
