# Supplementary material for: Non-random genomic integration - an intrinsic property of retrogenes in Drosophila?
Source: BMC Evol Biol. 2010 Apr 28;10:114. doi: 10.1186/1471-2148-10-114 (PMC2879276; doi:10.1186/1471-2148-10-114)
Supplement: Additional file 1 — CG4918 is a retroposed gene in Drosophila. [file 1471-2148-10-114-S1.DOC]

CG4918 is a retroposed in Drosophila:

From our previous SAGE, GLGI and RACE analysis (Metta et al. 2006; Metta and Schlötterer 2008), we observed two introns in the ortholog of the gene *RplP2* (CG4918) of *D. melanogaster* in *D. pseudoobscura*. These introns were present in the 5’ and 3’ UTR regions. We also observed presence of introns in *D. ananassae*, *D. pseudoobscura,* *D. persimilis*, *D.* willistoni, *D. virilis*, *D.* mojavensis and *D. grimshawi* (www.ncbi.nlm.nih.gov/dbEST/). In these species, the gene is located on Muller’s element A. However this gene lacks introns in *D. melanogaster*, *D. simulans*, *D*. *sechellia*, *D. yakuba* and *D. erecta*. In these species, the gene is located on the Muller’s element E. This suggests that a retroposition event has occurred in the ancestor leading to *D. melanogaster* species complex. We couldn’t find evidence for the existence of the parental copy in these species. Since the current gene prediction methods are heavily relying on the gene models of *D. melanogaster*, particularly coding DNA sequence, it is not possible to identify such retroposition events.

Metta, M., R. Gudavalli, J. M. Gibert, and C. Schlötterer. 2006. No accelerated rate of protein evolution in male-biased *Drosophila pseudoobscura* genes. Genetics **174**:411-420.

Metta, M., and C. Schlötterer. 2008. Male-biased genes are overrepresented among novel *Drosophila pseudoobscura* sex-biased genes. BMC Evol Biol **8**:182.

Supplementary table 1. BLASTP scores and E values indicating conservation of the candidate genes in other dipteran species spanning approximately 250 million years of divergence

|  | *Culex pipiens* | | *Aedes aegypti* | | *Anopheles gambiae* | |
| --- | --- | --- | --- | --- | --- | --- |
|  | Score | E value | Score | E value | Score | E value |
| CG11164 | 125.561 | 2.95E-27 | 120.553 | 9.73E-26 | 107.071 | 9.12E-22 |
| CG1354 | 377.481 | 4.41E-103 | 368.237 | 3.22E-100 | 380.178 | 8.67E-104 |
| CG14618 | 207.608 | 1.36E-59 | 220.32 | 7.83E-56 | 215.698 | 1.61E-54 |
| CG14779 | 86.2705 | 1.24E-33 | 165.236 | 2.13E-39 | 125.561 | 6.33E-70 |
| CG2059 | 100.14 | 6.69E-32 | 64.70 | 2.66E-17 | 114.78 | 4.50E-40 |
| CG2227 | 117.087 | 1.37E-37 | 166.78 | 6.99E-40 | 109.768 | 2.12E-37 |
| CG33250 | 226.868 | 8.82E-58 | 182.956 | 2.81E-63 | 229.18 | 1.49E-58 |
| CG8239 | 302.368 | 2.27E-80 | 276.944 | 8.63E-73 | 266.159 | 8.61E-83 |
| CG8939 | 593.964 | 8.50E-168 | 611.683 | 3.75E-173 | 597.816 | 6.72E-169 |
| CG9126 | 225.328 | 6.00E-80 | 290.041 | 4.43E-159 | 573.55 | 8.63E-162 |
| CG9172 | 312.77 | 5.18E-84 | 318.161 | 1.30E-85 | 315.464 | 9.29E-85 |
| CG9742 | 93.5893 | 4.59E-20 | 101.293 | 6.76E-21 | 104.375 | 5.82E-24 |
| CG6284 | 318.161 | 5.31E-100 | 334.724 | 3.20E-97 | 333.183 | 6.14E-95 |
| CG12375 | 162.925 | 6.52E-56 | 197.208 | 5.93E-49 | 204.142 | 4.49E-80 |
| CG4918 | 98.5969 | 4.38E-20 | 99.7525 | 2.00E-20 | 98.5969 | 4.49E-20 |
| CG5029 | 43.5134 | 0.012792 | 174.481 | 2.27E-50 | 172.94 | 1.90E-50 |
| CG11790 | 182.57 | 1.53E-44 | 123.635 | 1.83E-37 | 187.578 | 4.67E-46 |
| CG32441 | 145.591 | 1.73E-38 | 98.2117 | 2.33E-19 | 125.561 | 8.06E-31 |
| CG16771 | 315.464 | 4.05E-84 | 338.961 | 3.12E-91 | 291.967 | 2.32E-84 |
| CG14286 | 60.077 | 5.40E-08 | 63.929 | 3.68E-09 | 62.003 | 1.04E-11 |
| CG1639 | 211.075 | 6.13E-67 | 286.96 | 8.64E-77 | 269.626 | 1.46E-71 |
