# Supplementary material for: Non-random genomic integration - an intrinsic property of retrogenes in Drosophila?
Source: BMC Evol Biol. 2010 Apr 28;10:114. doi: 10.1186/1471-2148-10-114 (PMC2879276; doi:10.1186/1471-2148-10-114)
Supplement: Additional file 2 — Conservation of the candidate genes in other Dipteran species spanning approximately 250 million years of divergence. BLAST score and E-values of the genes in other Dipteran species based in default parameters in FlyBase. [file 1471-2148-10-114-S2.DOC]

Table: Conservation of the candidate genes in other dipteran species spanning approximately 250 million years of divergence

|  | *Culex pipiens* | | *Aedes aegypti* | | *Anopheles gambiae* | |
| --- | --- | --- | --- | --- | --- | --- |
|  | Score | E value | Score | E value | Score | E value |
| CG11164 | 125.561 | 2.95E-27 | 120.553 | 9.73E-26 | 107.071 | 9.12E-22 |
| CG1354 | 377.481 | 4.41E-103 | 368.237 | 3.22E-100 | 380.178 | 8.67E-104 |
| CG14618 | 207.608 | 1.36E-59 | 220.32 | 7.83E-56 | 215.698 | 1.61E-54 |
| CG14779 | 86.2705 | 1.24E-33 | 165.236 | 2.13E-39 | 125.561 | 6.33E-70 |
| CG2059 | 100.14 | 6.69E-32 | 64.70 | 2.66E-17 | 114.78 | 4.50E-40 |
| CG2227 | 117.087 | 1.37E-37 | 166.78 | 6.99E-40 | 109.768 | 2.12E-37 |
| CG33250 | 226.868 | 8.82E-58 | 182.956 | 2.81E-63 | 229.18 | 1.49E-58 |
| CG8239 | 302.368 | 2.27E-80 | 276.944 | 8.63E-73 | 266.159 | 8.61E-83 |
| CG8939 | 593.964 | 8.50E-168 | 611.683 | 3.75E-173 | 597.816 | 6.72E-169 |
| CG9126 | 225.328 | 6.00E-80 | 290.041 | 4.43E-159 | 573.55 | 8.63E-162 |
| CG9172 | 312.77 | 5.18E-84 | 318.161 | 1.30E-85 | 315.464 | 9.29E-85 |
| CG9742 | 93.5893 | 4.59E-20 | 101.293 | 6.76E-21 | 104.375 | 5.82E-24 |
| CG6284 | 318.161 | 5.31E-100 | 334.724 | 3.20E-97 | 333.183 | 6.14E-95 |
| CG12375 | 162.925 | 6.52E-56 | 197.208 | 5.93E-49 | 204.142 | 4.49E-80 |
| CG4918 | 98.5969 | 4.38E-20 | 99.7525 | 2.00E-20 | 98.5969 | 4.49E-20 |
| CG5029 | 43.5134 | 0.012792 | 174.481 | 2.27E-50 | 172.94 | 1.90E-50 |
| CG11790 | 182.57 | 1.53E-44 | 123.635 | 1.83E-37 | 187.578 | 4.67E-46 |
| CG32441 | 145.591 | 1.73E-38 | 98.2117 | 2.33E-19 | 125.561 | 8.06E-31 |
| CG16771 | 315.464 | 4.05E-84 | 338.961 | 3.12E-91 | 291.967 | 2.32E-84 |
| CG14286 | 60.077 | 5.40E-08 | 63.929 | 3.68E-09 | 62.003 | 1.04E-11 |
| CG1639 | 211.075 | 6.13E-67 | 286.96 | 8.64E-77 | 269.626 | 1.46E-71 |
