# Supplementary material for: Non-random genomic integration - an intrinsic property of retrogenes in Drosophila?
Source: BMC Evol Biol. 2010 Apr 28;10:114. doi: 10.1186/1471-2148-10-114 (PMC2879276; doi:10.1186/1471-2148-10-114)
Supplement: Additional file 3 — Likelihood ratio tests for branch models as implemented in PAML to test the evidence for an accelerated rate of evolution after retrotransposition (foreground lineage). [file 1471-2148-10-114-S3.DOC]

Table: Likelihood ratio tests for branch models as implemented in PAML to test the evidence for an accelerated rate of evolution after retrotransposition (foreground lineage)

|  | ω_F_ | ω_B_ | ln*ℓ*_0_ | ln*ℓ*_1_ | χ^2^ | p value |
| --- | --- | --- | --- | --- | --- | --- |
| CG11164 | 0.0941 | 0.0986 | -6319.88 | -6318.23 | 3.31 | 0.0690 |
| CG1354 | 0.0229 | 0.0195 | -4749.11 | -4749.00 | 0.22 | 0.6383 |
| CG14618 | 0.0416 | 0.0664 | -4933.62 | -4933.34 | 0.56 | 0.4548 |
| CG14779 | 0.0196 | 0.0236 | -3226.06 | -3226.00 | 0.13 | 0.7220 |
| CG2059 | 0.2146 | 0.109 | -5441.42 | -5439.44 | 3.95 | 0.0469 |
| CG2227 | 0.0016 | 0.1001 | -4811.93 | -4808.41 | 7.04 | 0.0080 |
| CG33250 | 0.0806 | 0.1463 | -5138.81 | -5137.57 | 2.49 | 0.1147 |
| CG8239 | 0.0935 | 0.1105 | -8003.12 | -8002.79 | 0.67 | 0.4146 |
| CG8939 | 0.071 | 0.0534 | -11008.25 | -11007.33 | 1.85 | 0.1741 |
| CG9126 | 0.0415 | 0.0426 | -6663.94 | -6663.93 | 0.01 | 0.9188 |
| CG9172 | 0.0877 | 0.0495 | -3531.23 | -3530.97 | 0.52 | 0.4710 |
| CG9742 | 0.0136 | 0.0057 | -803.33 | -802.95 | 0.76 | 0.3838 |
| CG6284 | 0.007 | 0.0649 | -5899.06 | -5898.30 | 1.52 | 0.2174 |
| CG12375 | 0.0441 | 0.0853 | -5105.24 | -5100.88 | 8.73 | 0.0031 |
| CG4918 | 0.0437 | 0.0717 | -1636.76 | -1636.44 | 0.65 | 0.4214 |
| CG5029 | 0.0349 | 0.0567 | -5625.71 | -5625.03 | 1.34 | 0.2470 |
| CG11790 | 0.5455 | 0.1208 | -5777.04 | -5775.31 | 3.46 | 0.0629 |
| CG32441 | 0.1044 | 0.0795 | -4011.06 | -4010.85 | 0.42 | 0.5169 |
| CG16771 | 0.059 | 0.0724 | -9006.80 | -9006.36 | 0.89 | 0.3455 |
| CG14286 | 0.0385 | 0.1178 | -3038.81 | -3036.83 | 3.95 | 0.0469 |
| CG1639 | - | - | - | - | - | - |

χ^2^ = 2Δ*ℓ* = 2(ln*ℓ*_1_ − ln*ℓ*_0_)

H_0_:  *ω*_B_ = ω_(FG)_

H_1_:  *ω*_B_ ≠ ω_(FG)_

*ω*_B_ means *ω* value for background and *ω*_F_ means *ω* value for foreground.

ln*ℓ*_0_ is likelihood for one ratio model

ln*ℓ*_1_ is likelihood for two ratio model

χ^2^ significance is calculated for 1 df. After bonferroni correction for multiple tests the significance of the p value is 0.0025.
